# LAMMER Kinase LkhA Plays Multiple Roles in the Vegetative Growth and Asexual and Sexual Development of *Aspergillus nidulans*

**DOI:** 10.1371/journal.pone.0058762

**Published:** 2013-03-13

**Authors:** Eun-Hye Kang, Ji-ae Kim, Hyun-Woo Oh, Hee-Moon Park

**Affiliations:** 1 Department of Microbiology and Molecular Biology, College of Bioscience and Biotechnology, Chungnam National University, Daejeon, Korea; 2 Industrial Bio-materials Research Center, Korea Research Institute of Bioscience and Biotechnology, Daejeon, Korea; University of Wisconsin – Madison, United States of America

## Abstract

LAMMER kinase plays pivotal roles in various physiological processes in eukaryotes; however, its function in filamentous fungi is not known. We performed molecular studies on the function of the *Aspergillus nidulans* LAMMER kinase, LkhA, and report its involvement in multiple developmental processes. The gene for LkhA was highly expressed during reproductive organ development, such as that of conidiophores and cleistothecia. During vegetative growth, the patterns of germ tube emergence and hyphal polarity were changed and septation was increased by *lkhA* deletion. Northern analyses showed that *lkhA* regulated the transcription of *brlA*, *csnD*, and *ppoA,* which supported the detrimental effect of *lkhA-*deletion on asexual and sexual differentiation. LkhA also affected expression of cyclin-dependent kinase NimX^cdc2^, a multiple cell cycle regulator, and StuA, an APSES family of fungal transcription factors that play pivotal roles in multiple differentiation processes. Here, for the first time, we present molecular evidence showing that LAMMER kinase is involved in *A. nidulans* development by modulating the expression of key regulators of developmental processes.

## Introduction

The LAMMER kinases are found throughout eukaryotes and are known to conserve the motif “EH**LAMMER**ILG”, which is important for the kinase activity and substrate recognition [1], [2]. These kinases possess dual-specificity kinase activity, resulting in the phosphorylation of serine, threonine, and tyrosine residues [1], [3]–[6]. Studies have reported that the LAMMER kinases are involved in cellular processes, including differentiation, cell cycle, stress response, and reproduction. Mutation of Doa, the LAMMER kinase in *Drosophila melanogaster*, results in recessive lethality [7], [8]. Furthermore, DOA regulates localization of TRA2, resulting in changes in the sex determination pathway [9]. The *Schizosaccharomyces pombe* LAMMER kinase, Lkh1, acts in filamentous adhesion growth on agar surfaces and negative regulation for a divalent cation-dependent asexual flocculation [10]. It phosphorylates an mRNA binding factor, Csx1, which is related to both oxidative stress response and sexual differentiation, and Tup transcription factors, which regulate expression of the glucose-repressible genes [11]–[13]. Recent results also indicate the involvement of Lkh1 in pre-mRNA splicing and cell wall assembly of the fission yeast [14], [15].

The homothallic ascomycete *Aspergillus nidulans* has a complex life cycle. The duplication cycle is initiated after breaking the dormancy of an asexual spore [16]. The cell then grows isotropically until first mitosis. After this process, direction of cell polarity is determined and the germ tube emerges. The elongating tip of this germ tube maintains the axis of polarity. The first septum is synthesized as early as the third nuclear division and the distance between septa is regular along the hyphae. The nuclei in the subapical compartment are arrested in interphase, whereas the nuclei in the apical tip of hypha continuously undergo mitosis.

During the asexual cycle, conidia (mitospores) reproduction is spatiotemporally controlled by a central regulatory pathway, which includes *brlA* and *abaA* [17]. BrlA, a key regulator of conidiophore development, controls the cessation of vegetative growth and initiation of asexual development [18]. *brlA* is expressed in the form of two overlapping transcriptional units, *brlAα* and *brlAβ*, that are required for normal conidiophore production [19]. AbaA is required for the differentiation and functionality of sterigmata (metulae and phialide) and is directly activated by BrlA [17]. Precise sequential expression of these genes is induced through *u*pstream *d*evelopmental *a*ctivators (UDAs), which are encoded by six genes (*fluG*, *flbA*, *flbB*, *flbC*, *flbD,* and *flbE*) that are responsible for vegetative growth, conidiation, sterigmatocystin biosynthesis, and trehalose biogenesis [20]. Aside from the central regulatory pathway, developmental modifiers, such as StuA, ensure the correct morphology of cellular differentiation [21], [22]. StuA, acting as a repressor during the asexual stage and activator during the sexual stage, affects normal formation of conidiophore pattern during cell differentiation, ascosporogenesis, and formation of the cleistothecial shell and Hülle cells [23].

Sexual reproduction is influenced by various environmental and endogenous factors; however, this process is not well characterized compare to the asexual cycle. Several factors affecting the maturation process of cleistothecia have been identified, including VeA, NsdD, COP9 signalosome (CSN), and psi factors (PpoA, B, and C) [24]–[31]. The CSN is a multi-protein complex composed by eight subunits, namely six PCI and two MPN domain proteins, in animals and plants [26]–[28]. However, some components of these CSN subunits are absent in fungi, except in *A. nidulans*. The fungal CSN complex is not essential for viability, but is involved in cell cycle progression and development. In addition, the CSN complex regulates the transcription level of *ppoA*, a psi (**p**recocious **s**exual **i**nducer) factor [31]. Psi factors (psi*β,* psi*α,* and psi*γ*) influence sexual and asexual sporulation, especially spatial modulation and balance between conidiation and ascosporogenesis [29]–[31].

As previously mentioned, differentiation in *A. nidulans* is controlled by complicated signal transduction and genetic regulatory pathways. Various developmental regulatory proteins are also involved in the formation of conidia during mitosis and ascospores during karyogamy (nuclear fusion) and meiosis. Cdc2, a mitosis-promoting factor (MPF), is universally conserved and central to the timing and initiation of mitosis in all eukaryotic cells [32]–[34]. In *A. nidulans*, the *nimX^cdc2AF^* mutant, which is mutated at the inhibitory phosphorylation sites Thr14 and Tyr15 into non-phosphorylatable residues, shows a deficiency in the S-phase and G2/M DNA damage checkpoint controls, resulting in defects in septation, sporulation, and morphogenesis during conidiation [35], [36]. A previous study reported that *nimX^cdc2^* mRNA expression is induced by ectopic expression of *brlA* [36]. Therefore, the interaction between developmental regulators and cell cycle factors affects morphogenesis in *A. nidulans*.

In this paper, we identified LAMMER kinase, a novel regulator of development, in filamentous fungi. *A. nidulans* LAMMER kinase, LkhA, was not essential for viability, but its deletion revealed pleiotropic phenotypes that show changes in the expression of a fungal specific APSES family consisting of a basic helix-loop-helix transcription factor, StuA. To our knowledge, this is the first study on the involvement of LAMMER kinase in developmental regulation and cell cycle progression in filamentous fungi.

## Materials and Methods

### Strains, media, and culture conditions

The *A. nidulans* strains employed in this study are listed in Table S1. The standard manipulation of *Escherichia coli* strain (DH5α) was applied as described elsewhere [37]. Standard fungal techniques for culture, observation, transformation, and genetic analyses were performed according to the previous reports [25], [38]. For phenotypic analyses, strains incubated for a given time at 37°C were used. Briefly, vegetative mycelia cultured for 14 h were transferred onto solid minimal media (MM) and incubated for asexual development. For oxygen-limiting (hypoxic) conditions, the vegetative mycelia were transferred to an MM plate containing 0.15% casamino acids, sealed with parafilm, wrapped with aluminum foil, and incubated for a given time. For sexual induction, after incubation under hypoxic conditions, the seals were removed after 24 h incubation and incubated further. Conidial suspensions were prepared using 0.08% Tween80 and conidia were counted using a hemocytometer.

### Nucleic acid manipulation and vector construction

Fungal genomic DNA was isolated as described by Kim *et al*. [39]. Primer pairs used in this study are listed in Table S2. For over-expression of genes, the open reading frame was amplified and inserted into the pNQ vector harboring a *niiA* promoter or pQa harboring an *alcA* promoter and *pyroA* marker for selection. The *niiA* promoter was induced using 0.6% sodium nitrate and repressed by adding 0.2% ammonium tartrate. The *alcA* promoter was induced by adding 2% threonine and repressed by adding 1% glucose.

### Construction of the *lkhA* deletion strain

Deletion mutant was created by the previous method [40]. The *Aspergillus nidulans argB* gene from FGSC4 was used as a selectable marker for transformation. The information of primers for fusion products are listed in Table S2. The fragments of deletion cassette for *lkhA* (each 1.2 Kb 5′- and 3′- flanking sequence, and *argB*) were amplified using primer sets, LkhA-A1/LkhA-A2, LkhA-B1/LkhA B2, and *argB*-For/*argB*-Rev. The full deletion cassette obtained by double joint PCR using nested primers LkhA-C1/LkhA -C2 (Table S2) was purified and used to transform TJ1. The homologous recombination was confirmed by Southern Blotting (data not shown).

### RNA preparation and Northern blotting

Total RNAs were prepared by a modified guanidine thiocyanate/CsCl density gradient ultracentrifugation method, as described by Glisin *et al.* [41]. Briefly, the cultured cells were harvested and ground to a powder with mortar, pestle, and liquid nitrogen. GT buffer (4 M guanidine thiocyanate, 25 mM sodium acetate (pH 6.0), 0.5% *N*-lauryl sarkosyl, 0.84% β-mercaptoethanol) was added to the cell powder. After vortexing, the debris was removed by centrifugation. The supernatant was then overlaid onto 2 ml of 5.7 M CsCl solution (5.7 M CsCl, 25 mM sodium acetate, pH 6.0). The RNA pellet was isolated by centrifugation at 10°C, 125,000×*g* for 16 h using an SW 55Ti rotor (Beckman Coulter, California, USA). The pellet was resuspended in 400 ul TES buffer (10 mM Tris-Cl, pH 7.4, 5 mM EDTA, 1% SDS) and extracted with phenol/chloroform (pH 4.5). RNAs were enriched by ethanol precipitation, dissolved in DEPC-treated water, and stored at −70°C. Ten to twenty micrograms of total RNAs preparations was separated on an 1% agarose gel containing formaldehyde and transferred onto a Hybond-N^+^ membrane (Amersham Biosciences, Buckinghamshire, UK). Gene-specific probes were prepared from PCR-generated fragments and labeled with [α-^32^P] dCTP using a random primer DNA labeling kit (Takara Bio, Shiga, Japan). Signal was visualized by exposing the filter to X-ray film (Kodak, Rochester, NY, USA). The band intensity was calculated by computer program Image J.

### Protein extraction and Western blotting

Protein extraction was performed as described previously [42]. Briefly, the cultured *A. nidulans* cells were harvested after an appropriate time and immediately ground using liquid nitrogen. The cells were resuspended in protein extraction buffer (50 mM Tris-HCl, pH 8, 150 mM NaCl, 1 mM EDTA, 1% NP-40) with 2 mM phenylmethylsulfonyl fluoride (PMSF), protease inhibitor cocktail (Merck, Darmstadt, Germany), 10 mM sodium fluoride and 1 mM sodium vanadate and centrifuged at 16,600×*g*, 4°C for 30 min to obtain the supernatant. One hundred and fifty micrograms of total protein was solubilized by boiling in 5x SDS-PAGE sample buffer [350 mM Tris-HCl, pH 6.8, 600 mM dithiothreitol, 36% glycerol, 350 mM sodium dodecyl sulfate, 0.012% (w/v) bromophenol blue] for 5 min and resolved on 12% SDS-PAGE gels. The proteins were electroblotted onto a PVDF membrane with transfer buffer (25 mM Tris-HCl, pH 8.8, and 192 mM glycine) using a Semiphor transfer unit (Hoefer, Inc., California, USA). The membrane was saturated with a blocking buffer [5% skim milk in TBST (20 mM Tris-HCl, pH 7.5, 30 mM NaCl, 0.05% Tween 20)] for at least 1 h at room temperature. The primary antibodies against the Cdc2 PSTAIRE motif (Santa Cruz Biotechnology) and α-tubulin (Sigma Chemical-Aldrich, Inc.), and secondary antibody against the goat anti-rabbit IgG-HRP (Santa Cruz Biotechnology) and goat anti-mouse IgG-HRP (Santa Cruz Biotechnology) were used. Immunoblotting was carried out by the enhanced chemiluminescent method using the WEST-one^TM^ Western Blot Detection System (iNtRON Biotechnology, Seongnam, Korea) as described in the manufacturer's manual.

### Microscopy

Calcofluor white and DAPI staining were performed as previously described [43]. Briefly, to stain the septa and nuclei, spores were inoculated on CM broth in a petri-dish, covered with a few coverslips and incubated for 1 day at 37°C. The coverslips were washed with phosphate-buffered saline 3 times and fixed in methanol and acetic acid (3:1 v/v) for 40 min. Calcofluor white solution (Fluorescent Brightener 28, F3543; Sigma-Aldrich) and DAPI (4′,6-diamidino-2-phenylindole, D9564; Sigma-Aldrich) were mixed, dropped onto the coverslips and incubated for 40 min. The stained cells were visualized by differential interference contrast (DIC) using an Olympus DP71 microscope digital camera (Olympus, Tokyo, Japan). For scanning electron microscopy, the samples were pre-fixed in 2.5% paraformaldehyde-glutaraldehyde mixture buffered with 0.1 M phosphate (pH 7.2) for 2 h, post-fixed in 1% osmium tetroxide in the same buffer for 1 h, dehydrated in graded ethanol, and then transferred to isoamyl acetate. The samples were then dried at the critical point in CO_2_. Finally, the samples were sputtered with gold in a SC502 ‘mini’ sputter coater (Quorum technology, West Sussex, UK) and observed using the scanning electron microscope (HITACHI S4300N; Hitachi High Technologies America, Inc., California, USA). For transmission electron microscopy, the cells were fixed in 2.5% paraformaldehyde-glutaraldehyde mixture buffered with 0.1 M phosphate (pH 7.2) for 2 h, post-fixed in 1% osmium tetroxide in the same buffer for 1 h, dehydrated in graded ethanol and propylene oxide, and embedded in Epon-812. Ultra-thin sections, prepared using a Leica ULTRACUT E ultramicrotome (Leica, Illinois, USA), were stained with uranyl acetate and lead citrate and examined under a CM-20 electron microscope (Philips Electron Optics, Eindhoven, Netherlands).

## Results

### The *lkhA* gene encodes a protein kinase with a LAMMER motif

**Figure 1:**
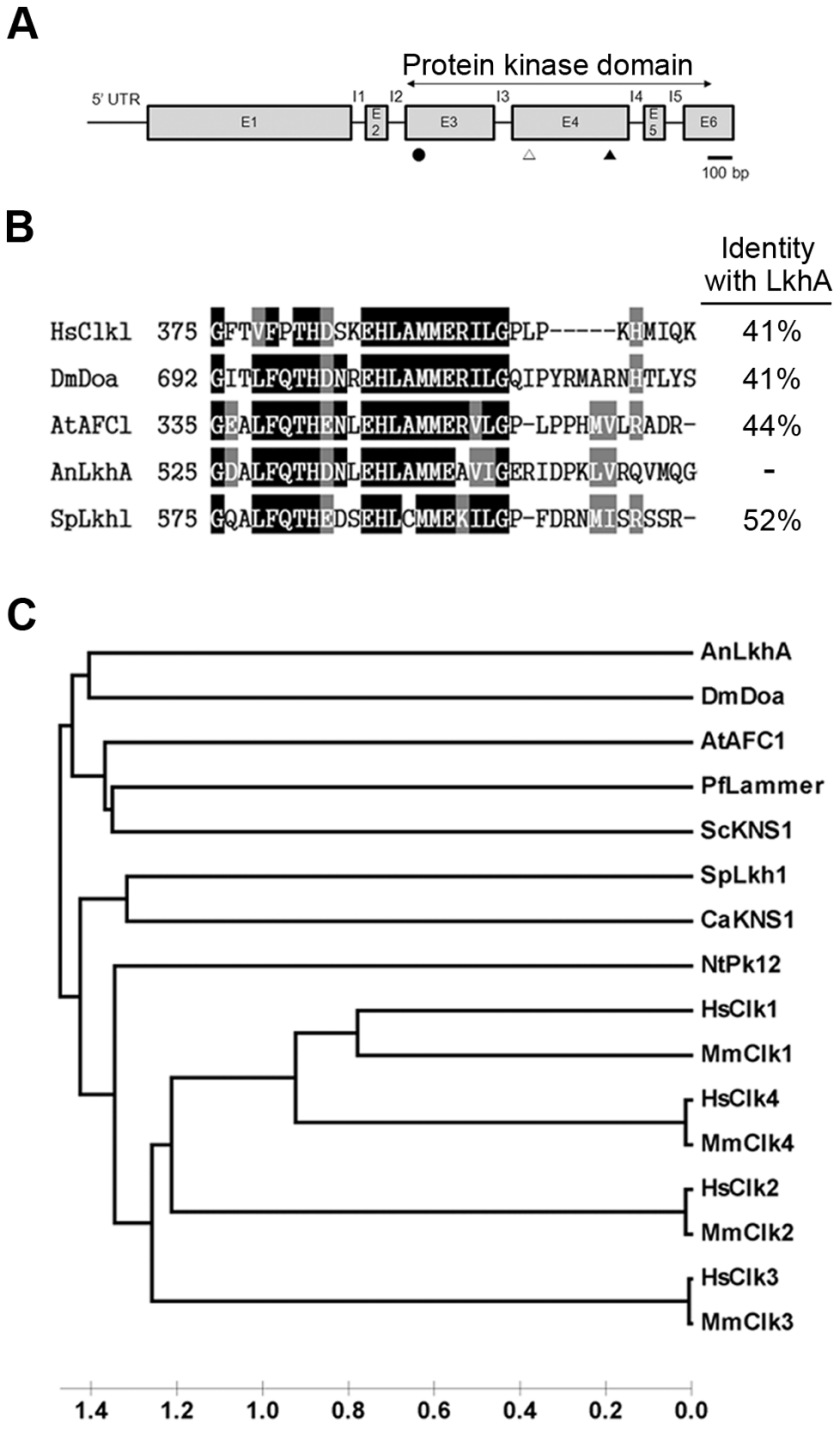
Analysis of *Aspergillus nidulans* LkhA sequence. **A**. A scheme of the protein kinase domain of *lkhA* was created using Scan Prosite. The black circle in the 3^rd^-exon indicates the ATP-binding region. The open and black triangles indicate the serine/threonine protein kinase active site signature and the LAMMER motif in the 4^th^ exon, respectively. The capital letters, E and I, represent exon and intron, respectively. **B.** Multiple alignment of amino acid sequences in LAMMER kinase orthologs created with Clustal-W. Black marks indicate identical amino acids. Gray marks indicate similar amino acids. **C**. Phylogenetic tree showing the evolutionary relationship of yeast to human LAMMER kinases. Phylogenies were inferred using MEGA 4.1 to create a neighbor-joining phylogenetic tree. Hs, *Homo sapiens*; Dm, *Drosophila melanogaster*; At, *Arabidopsis thaliana*; An, *Aspergillus nidulans*; Sp, *Schizosaccharomyces pombe*; Ca, *Candida albicans;* Sc, *Saccharomyces cerevisiae*; Pf, *Plasmodium falciparum*; Nt, *Nicotiana tabacum*; Mm, *Mus musculus*.

To understand the functions of LAMMER kinase in *Aspergillus nidulans*, a homologous gene was identified by searching through the *A. nidulans* genome database for its conserved motif, EH**LAMMER**ILG, and we named this LkhA (**L**AMMER **K**inase **H**omolog, Accession No. XM653500, AN0988). The ORF of *lkhA*, which was 2,253 bp in length, was divided into six exons and 5 introns (Figure 1A). The predicted LkhA protein had 667 amino acids and an estimated mass of 74.8 kDa. The catalytic domain of LkhA comprised the 3^rd^ to 6^th^ exons, which included an ATP-binding region in the 3^rd^ exon and a serine/threonine protein kinase active-site signature and LAMMER motif in the 4^th^ exon (Figure 1A). The predicted peptide sequence showed over 40% identity with other LAMMER kinases from various organisms. The LAMMER motif was highly conserved throughout yeast to humans, including *A. nidulans* (Figure 1B). Its phylogenic tree revealed that LkhA is the most closely related to Doa, the LAMMER kinase of *Drosophila melanogaster* (Figure 1C). These analyses also indicated that LkhA belongs to the family of LAMMER kinases.

### *lkhA* expression is reproductive stage specific

**Figure 2:**
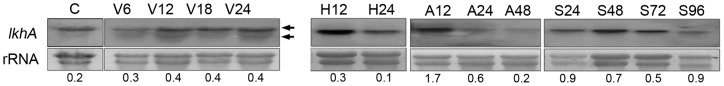
Expression of *lkhA* during development. Expression of *lkhA* mRNA is shown by Northern blot analysis with RNAs extracted from the wild type cells throughout life cycle. The capital letters, C, V, A, H, and S, indicate the conidia (C), vegetative stage (V), asexual development (A), hypoxic condition (H), and sexual development (S), respectively. Equal loading of RNA samples was evaluated by ethidium-bromide-stained rRNA bands. Numbers below the blots denote the relative density of each band normalized to the rRNA.

To analyze the temporal expression pattern of *lkhA*, the RNAs from the various developmental stages were subjected to Northern blot analysis (Figure 2). Northern blot analysis showed continuous *lkhA* expression throughout the *A. nidulans* life cycle, but the level of expression was relatively high at certain time points during asexual (A12) and sexual development (S24, S96). At least two isoforms of the *lkhA* transcript were detected during vegetative growth, suggesting that *lkhA* can influence fungal development, possibly through alternative splicing, which was earlier observed in *Drosophila* Doa.

### *lkhA* influences polarity and growth of vegetative hyphae

**Figure 3:**
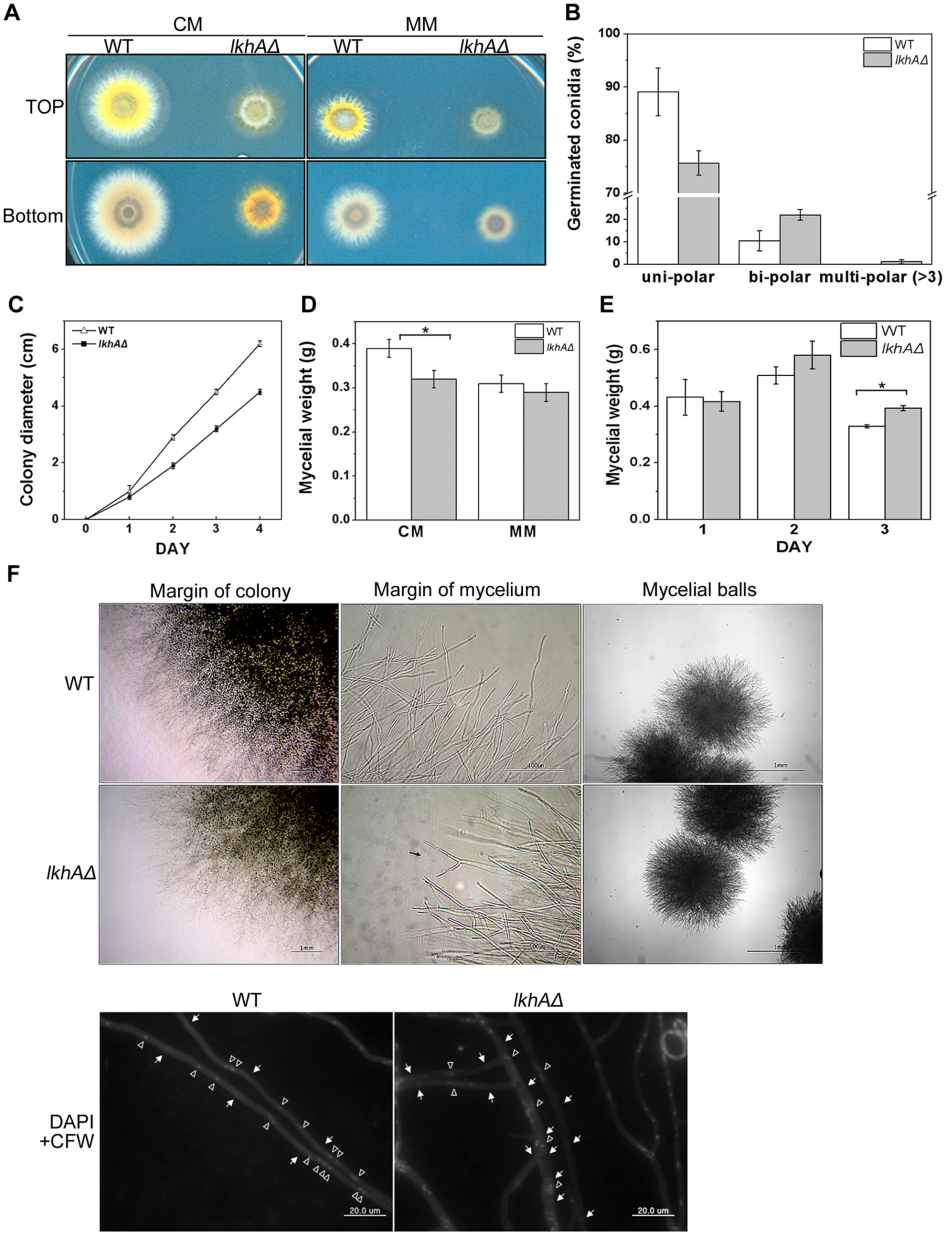
Growth pattern of the *lkhA* deletion strain. **A**. Colony morphology of wild type and *lkhAΔ* strain. **B**. Calculation of growth polarity. The strains were inoculated onto coverslips in CM, grown for 7 hrs, washed, and stained with Calcofluor white. The germlings from each strain were counted in triplicates, respectively. The P value of data is *<*0.04. **C**. Radial growth of wild-type and *lkhAΔ* strain. Over 4 days, the colony diameter of each culture from point-inoculated strains (inoculums, 10^6^ spores) was measured daily. **D**. Mycelial production on solid CM and MM. After incubation of point-inoculated strains for 3 days (inocula, 10^6^ spores) on a sheet of cellophane at 37°C, the weight of mycelial mass was measured. The P value of data from CM is *<*0.02. **E**. Mycelial production in liquid CM. After strains were cultured for 3 days at 37°C, mycelia were dried and the weight was measured. The P value of data from 3 days is *<*0.005. **F**. The pattern of branching, septation, and nuclear distribution in hyphae. Photos were taken with the colonies after 3 days on solid CM and the mycelial balls after 24 h in liquid CM, respectively. The conidia inoculated on coverslips were incubated to develop the hypha and the hyphae were stained by Calcofluor white and DAPI. The arrows and the open triangles indicate septa and nuclei, respectively.

To understand the functions of *lkhA*, a gene-deletion assay was performed by homologous recombination using *lkhA* knock-out cassette and confirmed by Southern analysis (data not shown). The *lkhAΔ* strain was viable, but showed abnormalities in growth such as reduced radial growth (Figure 3A) and deposition of reddish-yellow pigments in the agar culture medium (Figure 3A lower left panel). Because the *lkhAΔ* strain showed changes in polarity during germination, the proportion of germlings with isotropic and polar growth was calculated. The proportion of bipolar or mutipolar germlings was increased in the *lkhA* deletion mutants (Figure 3B), suggesting that the pattern of germ tube emergence and the maintenance of polarity axis were affected by LkhA. The colony diameter of *lkhAΔ* strains formed by spore point inoculation on agar after 1 day was 1.1 cm, whereas that in the wild type was 1.5 cm; the difference in colonial growth was increased with prolonged cultivation at 37°C (Figure 3C). Because the radial growth of deletion mutants was reduced, the extent of mycelium production was evaluated. As shown in Figure 3D, the *lkhAΔ* strain showed significant reduction in complete agar medium. The difference in mycelial production between wild type and *lkhAΔ* strain was not significant with 1 or 2 days cultivation, but was reversed and significant with prolonged cultivation (3 days) in liquid medium (Figure 3E), suggesting that disintegration (autolysis) of the mycelia might have been delayed by the *lkhA* deletion (See Figure S1). The hyphae from the marginal colonies of the *lkhAΔ* strain showed hyper-branching, whereas those of the wild type showed apical polarity during extension and moderate branching. The density of the mycelial ball was higher than that in the wild type in submerged culture (Figure 3F). To observe septation and nuclear distribution, staining with Calcofluor white (CFW) and 4,6-diamidino-2-phenylindole (DAPI) was performed (Figure 3F, lower-most panel). In wild type, cell compartments of vegetative hyphae were delimited by evenly placed septa and contained group of two or four nuclei. In the *lkhAΔ* strain, however, the septum interval was uneven and shorter than that in the wild type and the hyphae with frequent septation were thicker than those with normal septum intervals. Most interestingly, the *lkhAΔ* strain revealed numerical and morphological abnormalities in its nuclei, which were not punctuated but were instead aggregated in groups of varying number. These nuclear abnormalities were also found in the hyphae, and the septum intervals were in some cases similar to those in the wild type. These results indicate that *lkhA* affects regulatory mechanisms that are associated with the formation of the septum and its intervals and to the distribution of nuclei in accordance with the cell cycle. All of the mutant phenotypes observed in vegetative hyphae were reversed by the re-introduction of the *lkhA* gene into the deletion mutant (data not shown).

### *lkhA* affects conidiophore morphogenesis and conidia production

**Figure 4:**
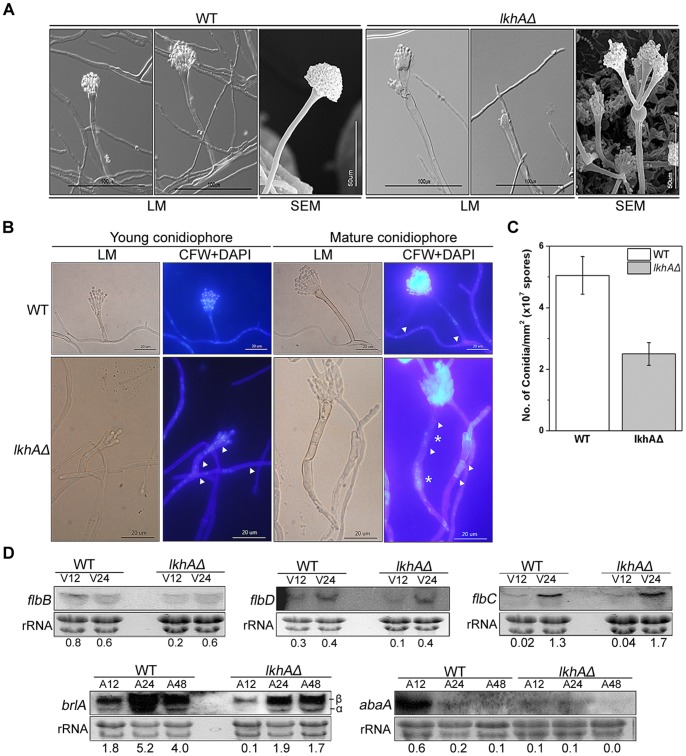
Effect of *lkhA*-deletion on an asexual development. **A**. Photographs of conidiophores showing abnormal morphology in the *lkhAΔ* strain under light microscope (LM) and scanning electron microscope (SEM). **B**. Calcofluor white and DAPI staining of asexual reproductive organs (conidiophores). The arrow heads indicate septa in stalk and stalk-like hyphae. The asterisks indicate nuclei in the stalks. **C.** Effect of the *lkhA*-deletion on conidia production (average of triplicate cultures with standard error, *P<0.01*). **D**. Expression level of transcription factors, *flbB*, *flbD*, *flbC*, *brlA*, and *abaA,* which affect morphogenesis of conidiophore. Numbers below the blots denote the relative density of each band normalized to the rRNA.

To investigate the function of LkhA in asexual development, the morphology of the asexual reproductive organ, the conidiophore, was examined using light (LM) and scanning electron microscopy (SEM). In contrast to normal conidiophores, which are highly symmetric structures consisting of a stalk, vesicle, double layer of sterigmatae (metulae and phialide), and a chain of dormant spores (conidia), those of the *lkhAΔ* strain displayed a reduced number of primary sterigmatae, the emergency of secondary conidiophores from its vesicle, and irregularity in the length of sterigmata (non-separated) (Figure 4A). When the formation and distribution of the septa and nuclei in conidiophores were examined (Figure 4B), the *lkhA* deletion mutants formed multiple septa in its stalk, whereas no septum is formed in the wild type, as well as abnormalities in nuclear behavior and a thick stalk similar to that in vegetative hyphae. The *lkhAΔ* strain also showed a lack of symmetry in sterigmata attributable to the absence of or just one phialide budding from a metula, in contrast to the wild type, which produces two phialides from a metula by mitosis. In addition, as shown in Figure 4C, the *lkhAΔ* strain produced half the number (∼53%) of conidia produced in the wild type.

To understand changes in asexual development at the molecular level that may be associated with the *lkhA* deletion, the expression of transcription factors for asexual development was analyzed. As shown in Figure 4D, the expression of upstream developmental activators, *flbB*, *flbC*, and *flbD,* showed no dramatic changes during the vegetative stage following by *lkhA* deletion. After the initiation of asexual development, however, the level of *brlAβ* and *brlAα* expression was dramatically decreased in the *lkhA* deletion mutants. The level of *abaA* expression was also reduced significantly in the *lkhA* deletion mutants approximately 12 h after asexual development. These results are consistent with abnormal conidiophore morphogenesis, particularly defects in sterigmata formation and reduced production of conidia in the *lkhAΔ* strain. These results suggest that the *lkhA* may play a role in conidiophore development through transcriptional modulation of *brlA* expression.

### *lkhA* regulates the expression of the cell division regulator NimX^Cdc2^

**Figure 5:**
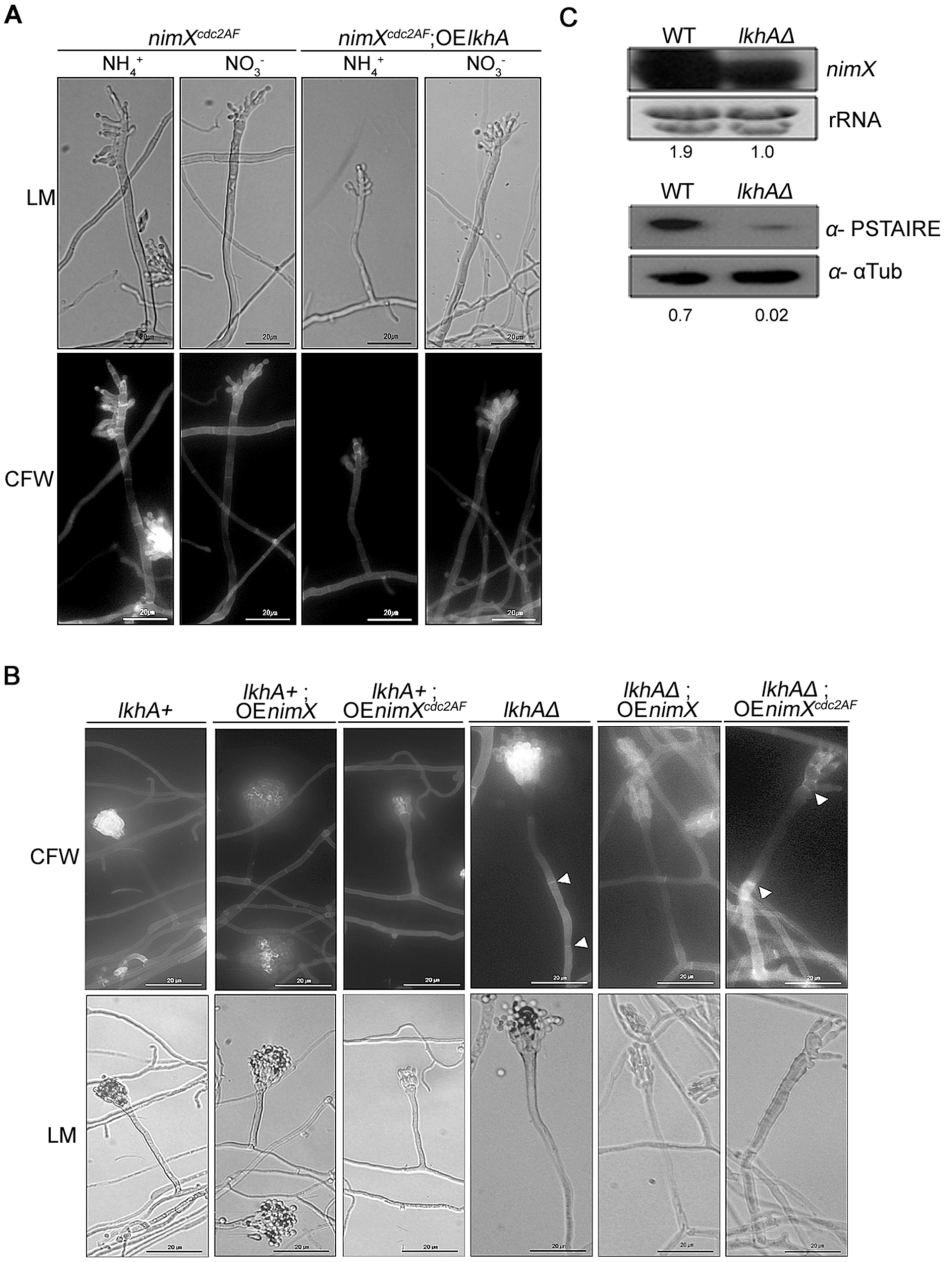
Relationship between LkhA and NimX^cdc2^. **A**. Over-expression of *lkhA* in *nimX^cdc2AF^* mutant. The strains were inoculated onto *nii*(p)-inducible media containing 6 mM hydroxyurea, incubated, and stained with Calcofluor white. **B**. Over-expression of *nimX* alleles in the wild type and *lkhA*-deletion strain. Cells were stained with Calcofluor white and the asexual reproductive organs (conidiophores) were examined. The arrow heads indicate septa in stalk. **C**. Expression level of mRNA (upper panel) and protein (bottom panel) of NimX^cdc2^. Numbers below the blots denote the relative density of the *nimX* transcripts normalized to the rRNA (upper panels) and those of NimX normalized to the α-tubulin (lower panels).

Interestingly, morphogenetic defects in conidiophores caused by *lkhA* deletion (Figure 4A, right panels) were similar to those of *nimX^cdc2AF^* mutants [36], which initiate mitosis under conditions of DNA replication inhibition, and are thus sensitive to hydroxyurea (HU) [35]. When *lkhA* was over-expressed by the control of the nitrate-inducible *niiA* promoter in the *nimX^cdc2AF^* mutant, there was no reversion of mutant phenotypes, such as sensitivity against HU (data not shown) and abnormal conidiophores morphogenesis (Figure 5A). Over-expression of *nimX* alleles in the wild type revealed no detectable changes in conidiophore morphogenesis (Figure 5B, 2^nd^ and 3^rd^ panels), whereas over-expression of *nimX* in the *lkhA*-deletion strain, a partial reversion was observed in that of the mutant phenotype, such as disappearance of septa in conidiophore stalk (Figure 5B, 5^th^ panel). Over-expression of *nimX^cdc2AF^* in the *lkhAΔ* strain resulted in the exacerbation of irregularities in conidiophores, including thickened and multi-septated stalks, lack of sterigmata (Figure 5B, right-most panel), and emergence of secondary conidiophores from the vesicle or metula (data not shown). In addition, Northern blot analysis of *lkhA* deletion mutants revealed a decrease in *nimX* transcription (Figure 5C, upper panel). Western blot analysis using anti-PSTAIRE antibody, which detects the conserved PSTAIRE domain in the Cdc2 family of kinases, also revealed a decrease in NimX translation (Figure 5C, lower panel). These results show that the *lkhA* deletion may have deleterious effects on vegetative growth and conidiophore morphogenesis based on changes in the cell division regulator NimX.

### *lkhA* affects cleistothecium maturation and ascospore production

**Figure 6:**
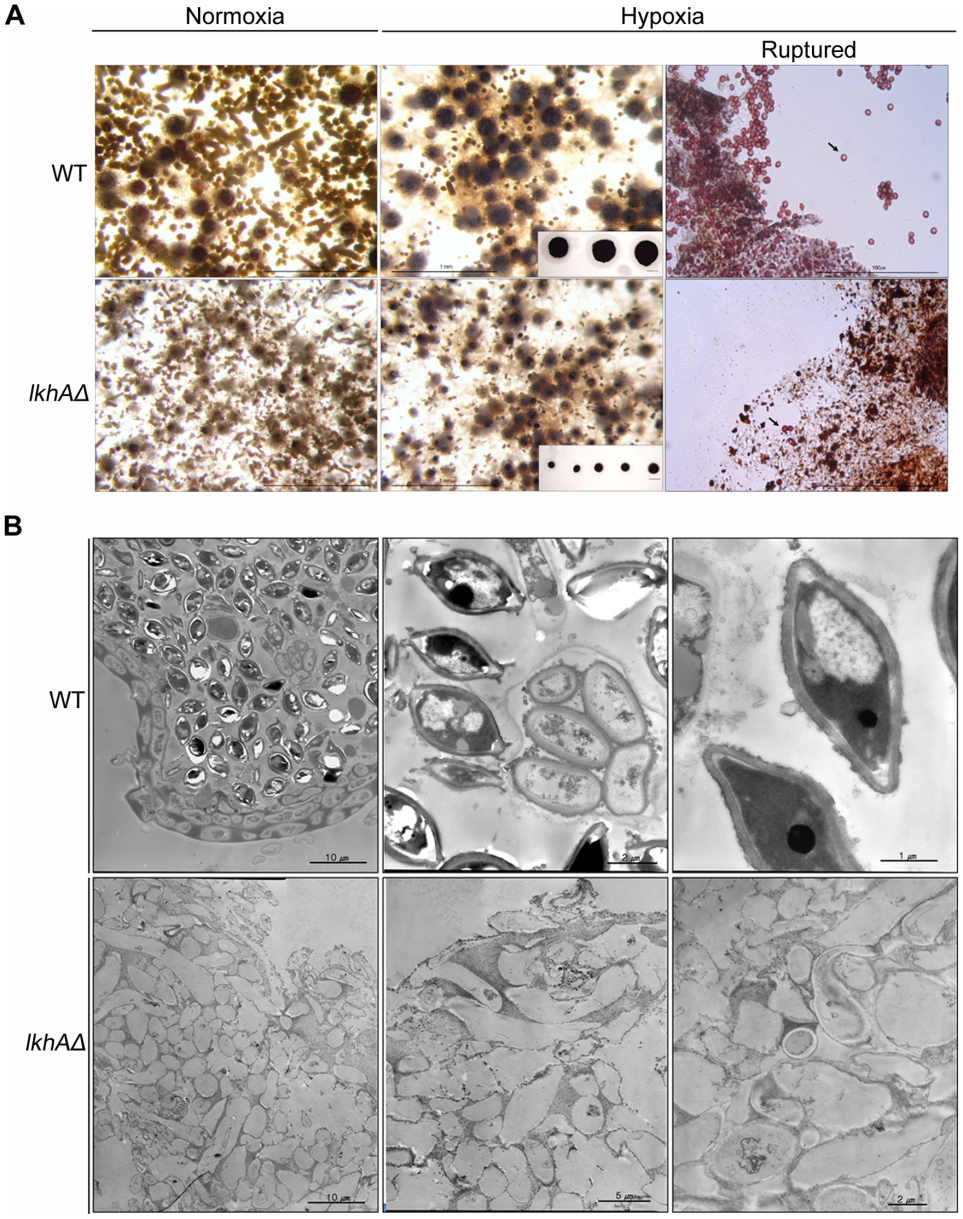
Effect of LkhA-deletion on sexual development. A. Development of cleistothecia in the wild type and the *lkhAΔ* strain. The black balls in the insert of middle panels are cleistothecia obtained after removing Hülle cells. The ruptured cleistothecia from the wild type and the *lkhAΔ* strain. Arrows indicates ascospores from cleistothecia (right panels). Scale bar indicates 20 µm. B. Interior of mature cleistothecia observed by transmission electron microscopy (TEM).

To determine the effects of LkhA on sexual differentiation, cleistothecium formation was investigated using cultures exposed to normoxic and hypoxic condition. The frequency of fruiting body formation revealed no significant changes regardless of sexual induction conditions and of the presence of *lkhA*, but the size of the cleistothecium was dramatically reduced in *lkhA* deletion mutants subjected to both conditions (Figure 6A, left and middle panels). When the cleistothecia formed under hypoxic conditions were collected and ruptured to examine the formation of ascospores, those from the wild type were fully mature and showed well-organized shells filled with numerous of reddish ascospores; however, those from the *lkhAΔ* strain were immature and showed more fragile shells filled with very few ascospores (Figure 6A, right panel). These results indicate that *lkhA* plays pivotal roles in sexual differentiation.

To observe the ultrastructure of the sexual organ, the cleistothecia collected at the late sexual stage were examined by transmission electron microscopy (TEM) (Figure 6B). Cleistothecia from the wild type fungi were filled with both fully matured ascospores and asci with developing ascospores and were enclosed within two well-developed peridial layers [44]. Moreover, the electron-dense substance, called “cleistin”, infiltrated only two outer layers, including the surface of peridial layer. On the other hand, the cleistothecia from the *lkhAΔ* strains were mostly filled with amorphous aggregates that resembled ascogenous cells and contained very few asci. The electron-dense cleistins were not restricted to the outer-most peridial layers, but infiltrated into the inner parts of the cleistothecia of the *lkhAΔ* strain. These results indicate that *lkhA* is required for the completion of cleistothecial development, including organization of the peridial layers and the ascogenous cells, which collectively comprise the process of ascosporogenesis.

**Figure 7:**
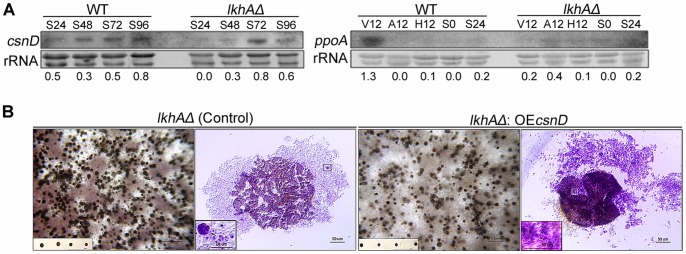
Effect of LkhA-deletion on expression of sexual genes. **A**. Expression of *csnD* and *ppoA* during vegetative (V), asexual (A), hypoxic (H), and sexual (S) developmental stage. Numbers below the blots denote the relative density of each band normalized to the rRNA. **B.** Effect of *csnD* over-expression in *lkhAΔ* strain on cleistothecia production. The black dots in the insert of the left panels are isolated cleistothecia and the photo in the insert of the right panels are the enlarged image of the region indicated with the small box in the background image.

To confirm whether the failure in formation of sexual reproductive organs was due to the changes in expression of the sexual reproductive organ-related genes, the transcription of sexual genes was analyzed. Because the developmental immaturity of cleistothecial primordia was observed by *lkhA* deletion, the transcription level of *csnD*, core component of the COP9 signalosome (CSN), which is particularly related with maturation of primordium to cleistothecium [27], was analyzed. On the basis of the immaturity of cleistothecia, the level of *csnD* transcription was lower, particularly during the early sexual stage (S24) of the *lkhAΔ* strain, whereas in the wild type a significant level of expression from the early stages to the late stages was observed (Figure 7A). Decrease in *csnD* transcription was also confirmed by qRT-PCR analysis using mRNAs from the cultures on solid medium, which allows asexual and sexual development (Figure S2). To test the relationship between *lkhA* and *csnD* in more detail, *csnD* was over-expressed under the control of the *niiA*-promoter in the *lkhAΔ* strain. Failures in development and maturation of the cleistothecial outer layers, such as toughness and pigmentation, were reversed by the over-expression of the *csnD*; however, no reversals were observed in terms of size of cleistothecia and the production of ascospores (Figure 7B). These results indicate that LkhA affects the maturation of cleistothecia from primordia not only by modulating *csnD* transcription but also by modulating other sexual gene(s). Because *lkhAΔ* showed a decrease in ascospore production, transcription of *ppoA*, which is involved in production of the psi factor, psiBα, was tested. The psiBα stimulates sexual spore production, but represses asexual spore production [31]. As shown in the right panel of Figure 6C, a decrease in *ppoA* transcription was observed at the vegetative stage (V12), whereas an upregulation of transcription was observed during sexual development in the wild type. These results suggest that *lkhA* influences ascosporogenesis through the transcriptional regulation of *ppoA*.

### *lkhA* deletion impairs the tight regulation of developmental processes

**Figure 8:**
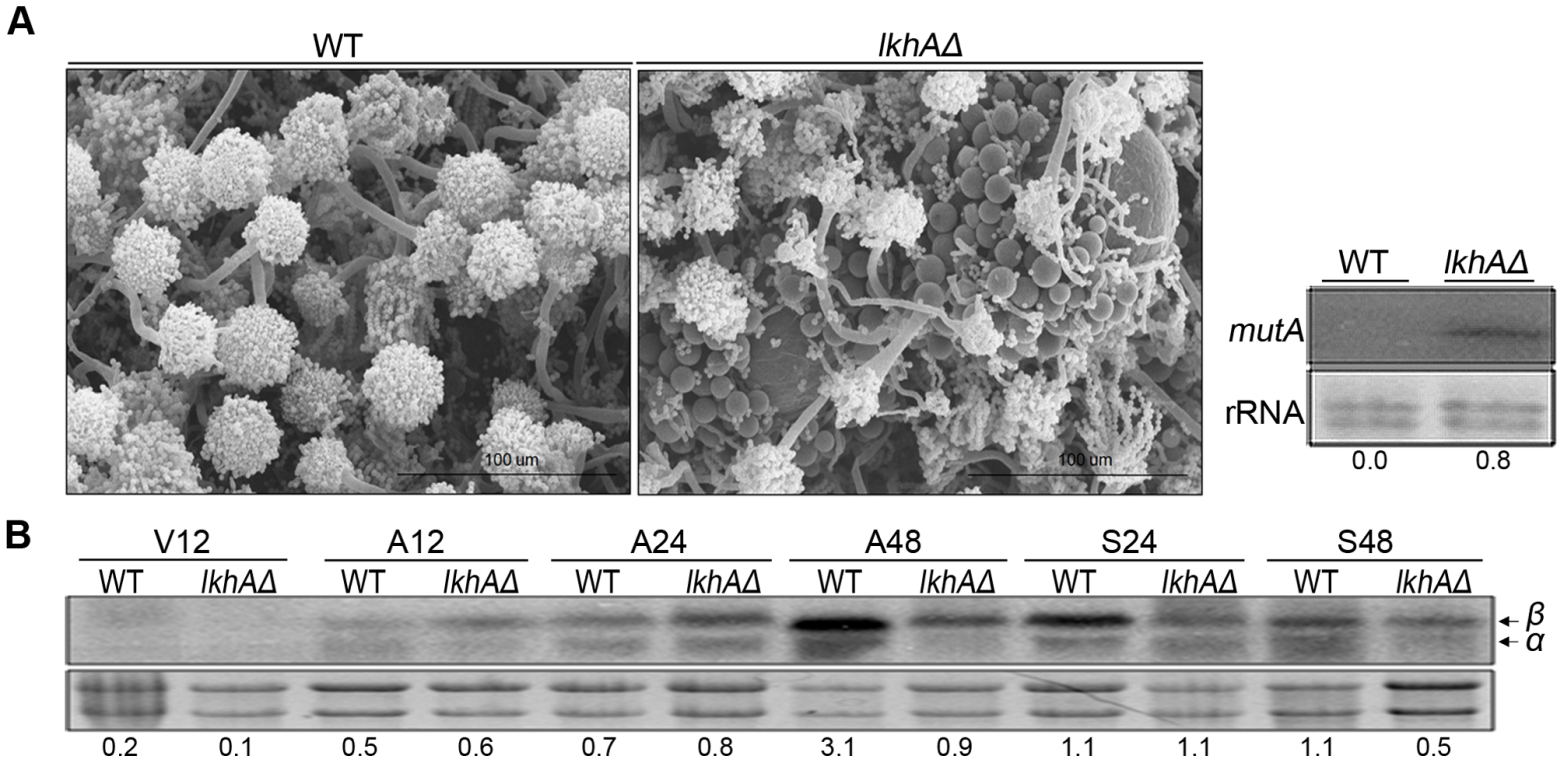
Effect of LkhA on temporal regulation for development. **A**. Formation of Hülle cells and cleistothecial primordia in the *lkhAΔ* strain at a time point when conidiophores exclusively appear in the wild type. The right panel shows the *mutA* transcript level detected by Northern blot analysis. **B**. Expression pattern of the *stuA* mRNAs, *stuAα* and *stuAβ*, during vegetative (V), asexual (A), and sexual (S) stages. Numbers below the blots denote the relative density of each band normalized to the rRNA.

The *lkhAΔ* strain cultured under normoxia showed an earlier appearance of Hülle cells (Figure 8A). The appearance of Hülle cells during cleistothecium development is coordinated with developmental stage [44]. Because sexual development requires massive cell cycle progression, α-1,3-glucanase (mutanase) degrades α-glucan to produce monosaccharides after the depletion of glucose. Because *mutA* encodes α-1,3-glucanase, which is localized in Hülle cells and connecting hyphae but not in other sexual organs of *A. nidulans*, its expression was examined to confirm the early appearance of Hülle cells [45]. In the *lkhAΔ* strain, expression of *mutA* was detected 48 h after asexual induction, whereas this was not observed in the wild type. The early appearance of sexual organs even under optimal conditions for asexual development in the *lkhAΔ* strain indicates that LkhA affects the temporal regulatory mechanism through the expression of development-specific genes in *A. nidulans*.

In *A. nidulans*, StuA affects the morphogenesis of conidiophores through the modulation of *brlA* and *abaA* expression, and *stuA* is also required for the formation of Hülle cells, cleistothecia, or ascospores [23]. To determine the effect of *lkhA* on *stuA* expression, total RNA was isolated from cultures at various developmental stages and subjected to Northern blot analysis (Figure 8B). In the *lkhA* deletion mutants, the expression levels of both *stuAα* and *stuAβ* were significantly decreased at 48 h during late asexual development, as well as at 48 h during sexual development compared to that in the wild type. These data suggest that LkhA modulates the temporal regulatory circuit for the differentiation of reproductive organs through *stuA* expression.

## Discussion

LAMMER kinases are involved in various cellular processes throughout yeast to human [7]–[14], [46]. In this study, we identified the gene for *A. nidulans* LAMMER kinase, LkhA, and provided evidence of its multiple roles in the development of a filamentous fungus.

### LkhA belongs to the LAMMER family and may play a role in development

*lkhA* follows a developmental stage-specific expression profile, which increases at certain time points during specific reproductive stages (A12, S24, and S48) and produces isoforms during the vegetative growth (Figure 2). This unique expression profile suggests that LkhA may play multiple roles in *A. nidulans* differentiation and reproduction. LkhA is phylogenetically related to the Doa protein of *D. melanogaster* (Figure 1C), which expresses at least six transcripts that have been generated through alternative splicing. For example, the 91 kDa isoform in the nerve system and the 69 kDa isoform in the head are expressed by Doa [47]. The occurrence of alternative transcripts of genes, such as *brlA*, *nsdC*, and *stuA*, which play pivotal roles in *A. nidulans* differentiation, is widely known [23], [39], [48]. Although its underlying mechanism remains unclear, the production of alternative *lkhA* mRNAs may reflect the organ- or stage-specific roles of LAMMER kinase during *A. nidulans* development

### LkhA affects the growth pattern of vegetative hyphae

When the effect of *lkhA* deletion on germ-tube formation and vegetative growth was analyzed, a significant reduction in the radial expansion of the colony (Figure 3A, C) and more frequent changes in polarity in germlings (Figure 3B) were observed. The mycelial yield in the *lkhAΔ* strain was higher than that observed in the wild type, even after prolonged cultivation in liquid culture (Figure 3E), indicating that the disintegration of mycelia in the *lkhAΔ* strain may be delayed (Figure S1). Fluorescent staining to visualize the septation and nuclear division in vegetative hyphae revealed abnormalities in the septum interval and nuclear distribution (Figure 3F), which may reflect the involvement of LkhA in cell division, similar to the LAMMER kinase in fission yeast [10], [46]. These results show that the activities of LkhA during vegetative growth are associated with the emergency of the germ-tube and the establishment and maintenance of polarity, apical growth (polarized extension), branching, septation, and nuclear division.

### LkhA is involved in the morphogenesis of the asexual reproductive organ

Upon completion of polar growth including the compartmentalization with septation and branching during vegetative growth, formation of multicellular reproductive structures occurs. Light and scanning electron microscopy have revealed extensive abnormalities during conidiophore development in the *lkhA* deletion mutants (Figure 4A). These abnormalities in the *lkhAΔ* strain were also associated with a reduction in conidia production (Figure 4C). As shown in vegetative hyphae, unusual formation of septa and abnormal nuclear distribution in the conidiophore stalk were also observed through fluorescence microscopy (Figure 4B) Therefore, these results indicate that LkhA may play multiple roles in the shape of conidiophores, including symmetry, septation in the stalk, the sequential development of sterigmata, and sporogenesis during asexual development. Although the expression level of genes for upstream developmental activators in asexual development, *flb*s, did not show any significant changes, the expression of *brlA,* a gene for key transcription factor of the central regulatory pathway in asexual development, was dramatically reduced in the *lkhA* deletion mutants (Figure 4D). Although the expression of both *brlAα* and *brlAβ* was decreased during asexual development, the overall decrease in *abaA* transcription in the *lkhAΔ* strain might have been mainly caused by a decrease in the number *brlAβ* transcripts. Because the activation of *brlAα* expression occurs through a positive feedback loop involving AbaA expression initiated by *brlAβ*, reduced *abaA* expression in the *lkhAΔ* strain may not be sufficient to support the increase in *brlAα* expression [22]. The results of mRNA analysis suggest that LkhA affects conidiophore morphogenesis by modulating the activity of unidentified upstream factor(s) other than the Flbs, which affect(s) the transcription of *brlA*. Moreover, the aberrant septum formation and the abnormal behavior of nuclei not only in conidiophore stalks but also in vegetative hyphae strongly indicate that LkhA is involved in the regulation of cell division.

### LkhA affects cell division by modulating the expression of NimX

In *A. nidulans*, development and cell division are closely connected; septum formation is dependent on the completion of the third nuclear division, which is characterized by the production of eight nuclei [49]. Further, the formation of septum is dependent on a signal generated from mitotic nuclei in the germ tube during cell extension and growth (cell size) [50]. Because abnormalities in nuclear distribution and septum formation caused by *lkhA* deletion (Figure 3F) were quite similar to those of NimX^cdc2AF^ mutants, the connection between NimX^cdc2^ and LkhA was investigated. Transcription (and thus translation) of NimX was reduced the *lkhA* deletion mutants (Figure 5C). The forced expression of *nimX* in the *lkhA* deletion mutants resulted in the partial reversal of phenotype i.e., disappearance of septa in the stalk (Figure 5B 5^th^ panel). This result suggests that LkhA affects the cell division cycle by modulating expression of the NimX^cdc2^. On the other hand, conidiophores of the *lkhAΔ* strain with forced expression of *nimX^cdc2AF^* (non-tyrosine phosphorylated form) showed an exacerbation of abnormalities, such as an increase in septa in the stalk and irregular formation of reproductive organs (Figure 5B, right-most panel). In yeast, the cell cycle is modulated by post-translational modifications of Cdc2/28 protein kinase, of which activity is regulated by the association with specific cyclins during the cell cycle. In higher organisms including humans, various CDKs (cyclin-dependent kinase) and cyclins control the cell cycle through transcriptional and post-translational modifications [51], [52]. In *A. nidulans*, the mechanism of cell cycle regulation by NimX^Cdc2^ has been extensively well-studied [35], [53]. During the cell division cycle, the phosphorylation of Tyr15 and Thr161 of NimX is important; Thr161-phosphorylation mediates the association of NimX and cyclinB, whereas Tyr15-phosphorylation blocks pre-mature mitosis, and Tyr15-dephosphorylation by Cdc25 allows entry into mitosis. The results presented here provide new insights into the regulation of fungal cell division by showing that LkhA affects the cell division cycle through the action of *nimX.*

### LkhA affects cleistothecial maturation by regulating transcription of a component of the COP9 signalosome and the psi factor

The COP9 signalosome (CSN) is associated with transcriptional regulation, protein phosphorylation, deubiquitination, development, DNA-damage checkpoint control by direct interaction with ataxia-telangiectasia mutated (ATM), and the cell cycle in mammals [54], [55]. In *A. nidulans*, it is involved in the control of light-dependent development, maturation of cleistothecia, hormone production, and secondary metabolism [26]–[28], [56]. In accordance with the severe defects in the maturation of cleistothecia (Figure 6A and B), *lkhA* – deletion led to a decrease in the transcription level of *csnD,* the gene that encodes a core component of the CSN complex, during the early sexual stage (Figure 7A). However, transcription of *csnE,* the gene for a regulatory component of the CSN complex was not affected by the *lkhA* – deletion (Figure S2). The defect in the maturation of cleistothecia in the *lkhAΔ* strain was partially suppressed by the over-expression of *csnD* (Figure 7B). Although further analyses are required, these results suggest that LkhA modulates CSN activity through *csnD*, but that LkhA may not be the only factor responsible for the CSN activity.

*ppoA* is involved in the production of a psi factor, psiBα, which balances the production of conidia and ascospores, including the initiation of asexual and sexual reproductive organs. The transcript level of *ppoA* in the *lkhAΔ* strain was reduced during acquisition of developmental competence (V12 in Figure 7A), resulting in a decrease in ascosporogenesis. Unlike the *ppoAΔ* mutant, which shows an increase in conidia production [31], the *lkhAΔ* strain showed a reduction in conidia production. This discrepancy may be attributable to the positive effect of reduced *ppoA* transcription on mitotic sporogenesis, which may have been masked by the defects in the asexual organ formation in the *lkhAΔ* strain. The reduction in mitotic sporogenesis was mainly caused by defects in conidiophore architecture, such as the reduced formation of sterigmata, and not by the reduction in conidia production per se.

It has been recently reported that LAMMER kinase is associated with sexual reproduction by phosphorylating Csx1, which binds to mRNA of *Ste11^+^*, a gene for the transcription factor in the sexual differentiation process in *S. pombe* [13]. The LAMMER kinase in fruit flies, DOA, is also known to play a role in somatic sex determination by phosphorylating SR-like proteins [57]. Although further investigation is required to understand the role of LAMMER kinase in the sexual development of eukaryotes, our results indicate that LkhA modulates the differentiation of sexual reproductive organs and ascosporogenesis by influencing the transcription of the COP9 component, *csnD*, and the psi factor, *ppoA.*

### LkhA is required for fine-tuning of the temporal regulation of development

Deletion of *lkhA* triggered the early development of sexual organs such as Hülle cells and cleistothecial primordial at the time points at which asexual organs are prevalent in the wild type (Figure 8A), suggesting the role of LkhA in fine-tuning fungal development. In the *lkhAΔ* strain, *mutA*, the indicator gene for development of Hülle cells, was expressed during late asexual development. This result indicates that *mutA*, which is repressed during asexual development, is not completely repressed in the *lkhAΔ* strain, and thus development of sexual organs would be triggered at an inappropriate time. Further, the profile of *stuA* transcripts (*stuAα* and *stuAβ*) was changed in *lkhA* deletion mutants (Figure 8B). StuA, a morphological modifier, determines the differentiation of reproductive organs by dictating the spatial organization of conidiophores, differentiation of sexual reproductive organs, and ascosporogenesis [23]. The transcriptional regulation of two *stuA* transcripts, *stuAα* and *stuAβ*, is complicated; these transcripts are initiated from different start sites and are alternatively spliced [23]. *stuAα* plays a significant role during both reproductive cycles, whereas *stuAβ* might contribute to the regulation of *stuA* expression. Although these transcripts encode an identical protein, the ratio of *stuAα* and *stuAβ* influences spatiotemporal differences in StuA localization. The variation in threshold requirements for each development based on a change in the *stuA* transcripts influences temporal patterns of differentiation in reproductive organs. In the *lkhAΔ* strain, the ratio of *stuA* transcripts was altered; *stuAα* was dramatically decreased during the late asexual (V48) and sexual stages (S48), indicating that LkhA may have regulated the factors that activate the transcription of *stuAα* and *stuAβ* at the upstream activation sequences (UASs) of *stuA.* These results suggest that LkhA directly and/or indirectly controls the expression of *stuA*, which is required for temporal regulation of the entire life cycle of *A. nidulans*.

### The pleiotropic phenotypes of LkhA deletion are caused by the transcriptional modulation of *brlA*, *stuA,* and cell cycle genes

**Figure 9:**
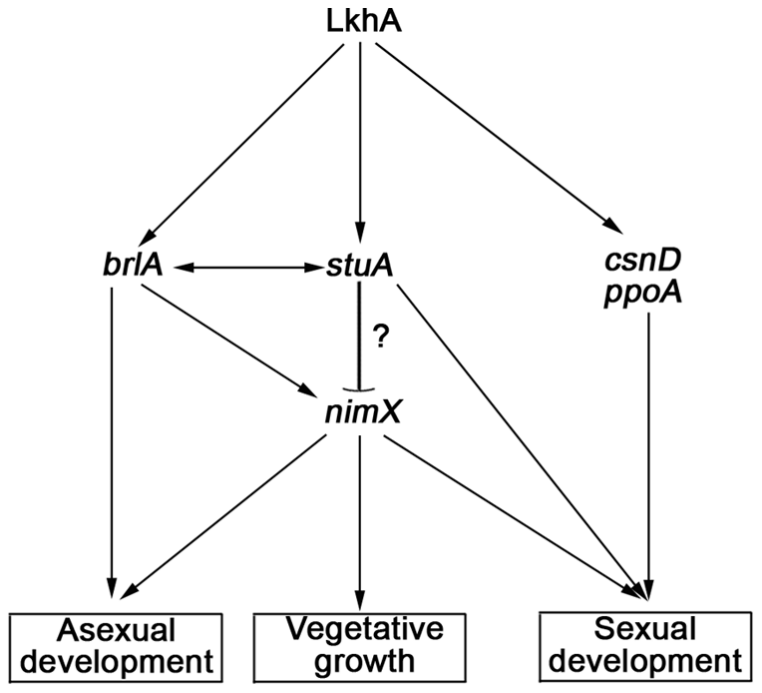
A proposed regulatory circuit for the LkhA-dependent differentiation of *Aspergillus nidulans*. Proposed interaction among the genes are indicated by the symbols (activation, →: repression/activation, –)).

The *lkhAΔ* strain showed pleiotropic defects in germination and formation of developmental organs. Northern blot analyses revealed that LkhA affected fungal differentiation through the transcriptional regulation of various developmental genes. Among these, *stuA* has recently been associated with morphogenesis, sporogenesis, pathogenicity, and the production of secondary metabolites in many filamentous fungi [58]–[61]. Although *stuAβ* may regulate *stuA* expression, *stuAα* is expressed in a *brlA*-dependent manner during development by binding to BrlA at the BrlA response elements found in the *stuAα* promoter [23]. Therefore, it is possible that LkhA regulates *stuA* expression through the transcriptional control of BrlA, particularly during mid- to late asexual development (Figure 8B; A48). It has been reported that the APSES proteins, including StuA, contain a sequence-specific DNA-binding domain with structural similarities to the eukaryotic basic helix-loop-helix (bHLH) protein [62] and that they control the critical G1/S phase cell-cycle transition in both *S. cerevisiae* and *S. pombe*. Interestingly, the sequence of StuA response elements (StREs), ^A^/_T_CGCG^T^/_A_N^A^/_C_, is identical to the MCB (*Mlu* I-cell cycle box) binding motif, ACGCGTNA, and the MBF (MCB-binding factor) complex that regulates the cell cycle in *S. cerevisiae* and *S. pombe*. In addition, the DNA-binding motif of StREs is enriched with promoter sequences of genes belonging to the functional MIPS category “Cell cycle and DNA processing” in the three ascomycetes, *Fusarium graminearum*, *Neurospora crassa*, and *S. cerevisiae* [63]. Previously, the existence of StREs upstream of the *A. nidulans* cell cycle genes *nimE* and *nimO* was reported [62], and sequence analysis of the *nimX* promoter region also revealed two StREs located at −434 bp and −444 bp (data not shown). These results suggest that the transcription of *nimX* is modulated by StuA. Although further investigation is needed, it may be postulated that LkhA modulates the regulatory circuit, which consists of BrlA, StuA, and cell cycle genes, including NimX (Figure 9).

In summary, this study presents several pieces of evidence, supporting the connection that LkhA is not essential for viability, but is involved in germ-tube development, maintenance of axis polarity in germlings, septation in conjunction with cell division cycle, and development of reproductive organs, such as conidiophores and cleistothecia, during the life cycle of the filamentous fungus, *A. nidulans*. These pleiotropic effects of LkhA in *A. nidulans* differentiation are mainly attributed to the sequential and concerted actions of BrlA, StuA, and NimX during asexual development and of CsnD and PpoA during sexual development. The results presented here will provide new insights into the molecular mechanisms underlying development in *A. nidulans*.

## Supporting Information

Figure S1 **Morphology of mycelial balls in submerged culture.** Strains were shaking-cultured for 3 days in Erlenmeyer flask containing liquid medium. The cultures were poured into a petri-dish and the mycelial balls were photographed.
(TIF)

Figure S2 **qRT-PCR analysis of mRNA level for** ***csnE*** **and** ***csnD*** **, which are components of the COP9 signalosome.** Relative mRNA levels were determined by quantitative real-time (qRT)-PCR. Gene expression levels were normalized with *tubC* amplified RNAs extracted from 14 h cultured mycelia. The X-axis indicates the culture time after transfer of mycelia onto the MM to allow development.
(TIF)

Table S1 **List of** ***A. nidulans*** **strains used in this study.**
(DOCX)

Table S2 **List of oligonucleotides used in this study.**
(DOCX)
