# Supplementary figures and images for: LAMMER Kinase LkhA Plays Multiple Roles in the Vegetative Growth and Asexual and Sexual Development of Aspergillus nidulans
Source: PLoS One. 2013 Mar 13;8(3):e58762. doi: 10.1371/journal.pone.0058762 (PMC3596290; doi:10.1371/journal.pone.0058762)

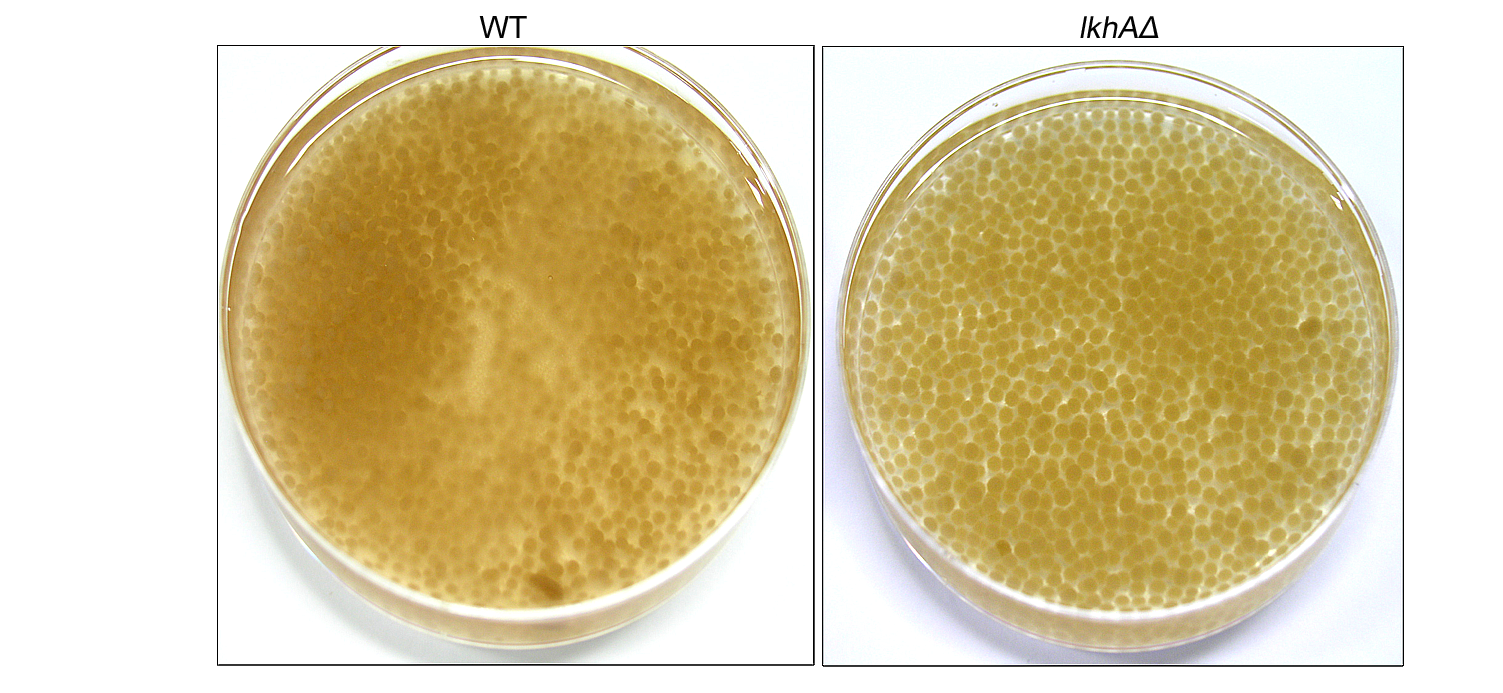

Supplement: Figure S1 — Morphology of mycelial balls in submerged culture. Strains were shaking-cultured for 3 days in Erlenmeyer flask containing liquid medium. The cultures were poured into a petri-dish and the mycelial balls were photographed. (TIF) [file pone.0058762.s001.tif]

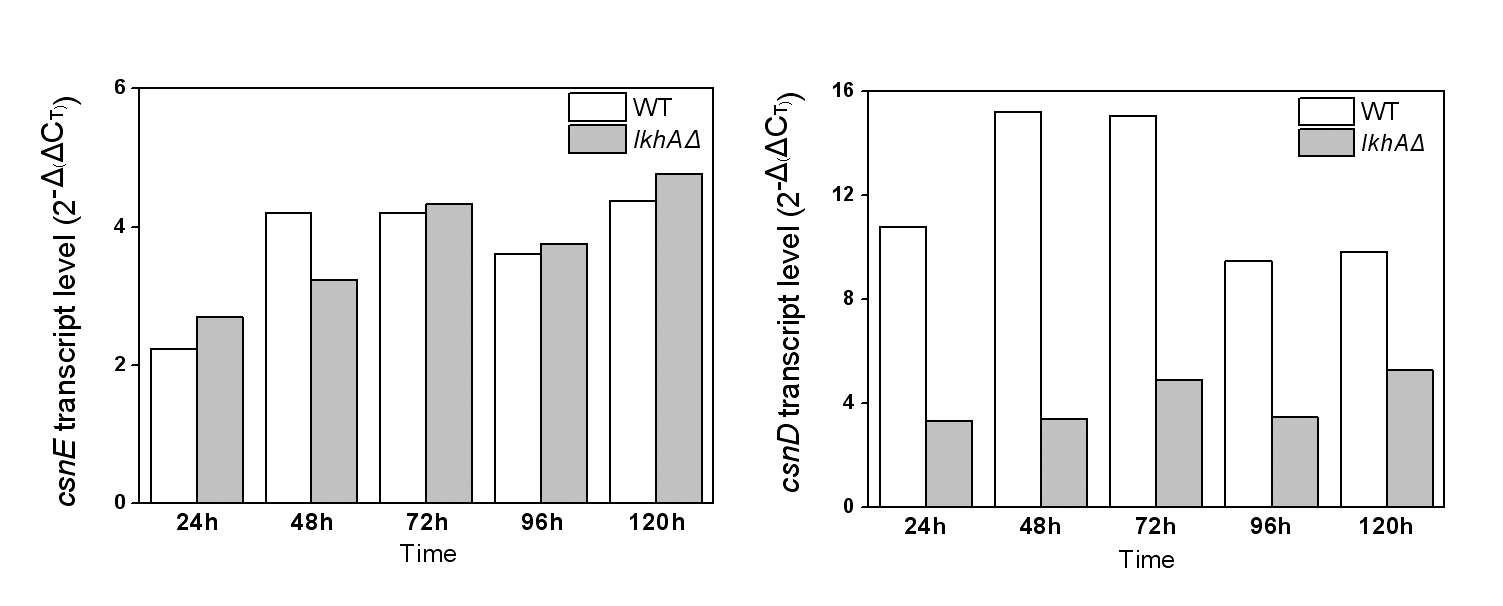

Supplement: Figure S2 — qRT-PCR analysis of mRNA level for csnE and csnD , which are components of the COP9 signalosome. Relative mRNA levels were determined by quantitative real-time (qRT)-PCR. Gene expression levels were normalized with tubC amplified RNAs extracted from 14 h cultured mycelia. The X-axis indicates the culture time after transfer of mycelia onto the MM to allow development. (TIF) [file pone.0058762.s002.tif]
