# Supplementary material for: LAMMER Kinase LkhA Plays Multiple Roles in the Vegetative Growth and Asexual and Sexual Development of Aspergillus nidulans
Source: PLoS One. 2013 Mar 13;8(3):e58762. doi: 10.1371/journal.pone.0058762 (PMC3596290; doi:10.1371/journal.pone.0058762)
Supplement: Table S1 — List of A. nidulans strains used in this study. (DOCX) [file pone.0058762.s003.docx]

**Table S1. List of *A. nidulans* strains used in this study**

| Strains | Genotype | Sources |
| --- | --- | --- |
| FGSC4 | *veA+* | FGSC^a^ |
| TJ1 | *yA2*; *argB2*; *pyroA4*; *veA+* | S. K. Chae |
| SK880 | *yA2*; *pyroA4*; *veA+;pILJ16 (argB+)* | S. K. Chae |
| DLA1 | *yA2*; *argB2*; *pyroA4; veA+;* ***ΔlkhA::argB*** | This study |
| DLAO1 | *yA2*; *argB2*; *pyroA4; veA+;* ***ΔlkhA::argB; nii(p)::pyroA*** | This study |
| DLACO1 | *yA2*; *argB2*; *pyroA4; veA+;* ***ΔlkhA::argB; nii(p)::csnD::pyroA*** | This study |
| FRY20 | ***nimX + nimX^cd2AF^****; pyroA4; pyrG89; pyr4+; wA3* | S.A. Osmani |
| FRY20-1 | ***nimX^cd2AF^****; pyroA4; pyrG89; pyr4+; wA3* | This study |
| FRY20-1-1 | ***nimX^cd2AF^****; pyroA4; pyrG89; pyr4+; wA3;* ***nii(p)::pyroA*** | This study |
| FRY20-1-L1 | ***nimX^cd2AF^****; pyroA4; pyrG89; pyr4+; wA3;* ***nii(p)::lkhA::pyroA*** | This study |
| SK880-O2 | *yA2*; *pyroA4*; *veA+****; alcA(p)::pyroA*** | This study |
| SK880-NO2 | *yA2*; *pyroA4*; *veA+****; alcA(p)::nimX::pyroA*** | This study |
| SK880-NAFO2 | *yA2*; *pyroA4*; *veA+;* ***alcA(p)::nimX^cdc2AF^::pyroA*** | This study |
| DLAO2 | *yA2*; *argB2*; *pyroA4; veA+;* ***ΔlkhA::argB; alcA(p)::pyroA*** | This study |
| DLANO2 | *yA2*; *argB2*; *pyroA4; veA+;* ***ΔlkhA::argB; alcA(p)::nimX::pyroA*** | This study |
| DLANAFO2 | *yA2*; *argB2*; *pyroA4; veA+;* ***ΔlkhA::argB; alcA(p)::nimX^cdc2AF^::pyroA*** | This study |
| SSNI58 | *ΔargB::trpCΔB*; *pyroA4*; *veA1*; ***NimX: :FLAG*** | R. Fisher |
| SSNI58DLA1 | *ΔargB::trpCΔB*; *pyroA4*; *veA1*; ***NimX: :FLAG****;* ***ΔlkhA::argB*** | This study |
| ^a.^ Fungal Genetics Stock Center, Kansas City, KN, USA. | |  |
