# Supplementary material for: LAMMER Kinase LkhA Plays Multiple Roles in the Vegetative Growth and Asexual and Sexual Development of Aspergillus nidulans
Source: PLoS One. 2013 Mar 13;8(3):e58762. doi: 10.1371/journal.pone.0058762 (PMC3596290; doi:10.1371/journal.pone.0058762)
Supplement: Table S2 — List of oligonucleotides used in this study. (DOCX) [file pone.0058762.s004.docx]

**Table S2. List of oligonucleotides used in this study**

| Oligo | Sequence (5' - 3') | Gene (locus) |
| --- | --- | --- |
|  | | |
| ***lkhA* disruption and complementation** | | |
| LkhA-A1 | GCA TGG ATT CAT GGG CCA TCT T | *lkhA* |
| LkhA-A2 | **AGT CAA ATG AGG CCT CTA AAC** TGG TCA GAT GGT TTA GAG AAA CCG | *lkhA* |
|  |  |  |
| LkhA-B1 | **AGC CAA GGT AGA TCC AGG CCT AAC ACA**  GCA TTC TAG CCT GAG TCC | *lkhA* |
|  |  |  |
| LkhA-B2 | ACT ACG GAC ACT TAC CCC TTT A | *lkhA* |
| *argB*-For | GAC CAG TTT AGA GGC CTC | *argB* |
| *argB*-Rev | GTG TTA GGC CTG GAT CTA | *argB* |
| LkhA-C1 | GCA TGG ATT CAT GGG CCA TCT T | *lkhA* |
| LkhA-C2 | AGG AGA AAT GGT TCT GCT TCG C | *lkhA* |
|  |  |  |
| **For Northern probe** | | |
| LkhA-E1 | ATG TCT ACA CCC TCA ACT | *lkhA* |
| LkhA-E2 | TCG ATC AGT GAT AGG GGT | *lkhA* |
| *flbB-*For | AAC AAC ATG TTG AAA GAG ATC CTA G | *flbB* |
| *flbB-*Rev | TTG TCG GCT AAG ATT GAG TAG CGT | *flbB* |
| *flbD-*For | CCC CAC GGC CGC ATC GAA GCT C | *flbD* |
| *flbD-*Rev | ACG GGA GGA AGA GAG TCG TGT GA | *flbD* |
| *flbC-*For | ATG ACG ATG GTT ATT GAG AAC CAG A | *flbC* |
| *flbC-*Rev | GTT AGA GAC AAC GGA AAA GTG TCG | *flbC* |
| *brlA-*For | ATG CGA AAT CAG TCC AGC CTG TCC | *brlA* |
| *brlA-*Rev | TGG CAA CAT GTT ATT CAT TGA CAT | *brlA* |
| *abaA-*For | ATG GCT ACT GAC TGG CAA CC | *abaA* |
| *abaA-*Rev | AAC CCA TAG GAG GAA AGT CA | *abaA* |
| *stuA-*For | ATG GCC AGC ATG AAT CAA CCT CAA | *stuA* |
| *stuA-*Rev | AAC GCC CTT CAG ATG CAT CGG GCC | *stuA* |
| *mutA-*For | GTT GGC ATC GTC GAA AAC TAT ACC | *mutA* |
| *mutA-*Rev | TCA ATC GAT GTC GTC GCG TG | *mutA* |
| *csnD-*For | CTC CGA AAC CGT GTC GGC ATC T | *csnD* |
| *csnD-*Rev | GCC GAA TTC TTC AAC GGA GGT C | *csnD* |
| *ppoA-*For | GAT AAT TCC GTG TCC AAG ACA TCG | *ppoA* |
| *ppoA-*Rev | CAT CGT CGT TGA ATG CAC CGT C | *ppoA* |
|  |  |  |
| **For over-expression** | | |
| LkhA-D1 | **GGA TCC** ATG TCT ACA CCC TC | *lkhA* (***Bam*H I**-tail) |
| LkhA-D2 | TCA ACG CTG TTG ACC GTT | *lkhA* |
| *csnD*OE*-*For | ATG CCA TCC CAA AAG ATA ATC TCC | *csnD* |
| *csnD*OE*-*Rev | **AAG CTT** TCA ACG TAC CAG ATG G | *csnD* (***Hin*d III**-tail) |
| *nimX*OE*-*For | **CTG CAG** ATG GAA AAC TAC CA | *nimX, nimX^cdc2AF^* (***Pst* I**-tail) |
| *nimX*OE*-13His-*Rev | **AAG CTT** TTA (GTG)_13_ AAA GCC ATT GCG GCG AGC GCG A | *nimX, nimX^cdc2AF^  (****Hind* III***-tail)* |
|  |  |  |
